# Nitrogen Adsorption and Characteristics of Iron, Cobalt, and Nickel Oxides Impregnated on SBA-15 Mesoporous Silica

**DOI:** 10.3390/nano13061015

**Published:** 2023-03-11

**Authors:** Jiun-Horng Tsai, Ting-Yi Lee, Hung-Lung Chiang

**Affiliations:** 1 Department of Environmental Engineering, Research Center for Climate Change and Environment Quality, National Cheng Kung University, Tainan 70101, Taiwan; 2 Department of Health Risk Management, China Medical University, Taichung 40402, Taiwan; 3 Department of Safety Health and Environmental Engineering, National Yunlin University of Science and Technology, Yunlin 640301, Taiwan; 4 Department of Occupational Safety and Health, China Medical University, Taichung 40402, Taiwan

**Keywords:** Santa Barbara Amorphous-15 (SBA-15), iron, cobalt, nickel

## Abstract

Hexagonal SBA-15 mesoporous material was used as a catalytic template for impregnation, with the transition metals Fe, Co, and Ni as catalysts for chemical transformation. Nitrogen adsorption/desorption isotherms, scanning electron microscopy, and transmission electron microscopy were conducted to better understand the physicochemical properties of the metal oxide-impregnated SBA-15. The specific surface area of the original SBA-15 was approximately 680 m^2^/g, and the abundances of the catalysts impregnated ranged from 2 to 8%, corresponding to specific surface areas of 560–470 m^2^/g for Fe-SBA-15, 440–340 m^2^/g for Ni-SBA-15, and 410–340 m^2^/g for Co-SBA-15. The increase in impregnated metal loadings filled the pores and collapsed the silica walls during the metal oxides impregnation on SBA-15 and calcination procedures, resulting in a decrease in the specific surface area and pore volume of the templates. The results showed that the order of nitrogen adsorbed was SBA-15 > Fe-SBA-15 > Ni-SBA-15 > Co-SBA-15 when the metal loading was 5%. In addition, the metal oxides on SBA-15 increased the wall thickness compared with raw SBA-15. Based on the XRD spectrum analysis, Fe_2_O_3_, Co_3_O_4_, and NiO were the stable crystals on the Fe-SBA-15, Co-SBA-15, and Ni-SBA-15, respectively. The sequence of the average grain size of metal oxides on SBA-15 was Co-SBA-15 > Fe-SBA-15 > Ni-SBA-15, according to XRD spectra and Scherrer’s equation. Isopropanol could be decomposed by metal oxide-impregnated SBA-15 to form carbon filament materials. Therefore, these materials have the potential to be employed for pollutant removal, catalytic reactions for organic solvent and bio-oil/biomass reforming, and recycling waste into high-value materials.

## 1. Introduction

SBA-15, an ordered mesoporous silica material, has hexagonally mesoporous structures, a high surface area, a uniform pore size, and tubular pore topologies, all of which are characterized by structural ordering over a long distance [1,2].The highly suitable properties of the material have been widely applied as catalysts and catalyst supports to convert hydrocarbons, adsorbents for separation, adsorption for a different medium, as energy storage for substances such as hydrogen, and as sensors for detection and monitoring.

According to the physicochemical properties of highly ordered and uniform nanoscale hexagonal and cylindrical mesopores, and the chemical and thermal stability of SBA mesoporous silica materials, they are more hydrothermally stable due to their thickness and condensed silica walls that strengthen the structure [1,3]. In addition, the inherent textural and morphological properties of the SBA-15 template enhance the dispersion of catalysts, revealing high efficiency for hydrocarbon conversions [4]. SBA-15 mesoporous materials exhibit high mass transport and diffusion characteristics and CO conversion in Fischer−Tropsch synthesis reactions [5], and convert CO_2_toCO [6]. Therefore, mesoporous SBA-15 could be suited as hard templates during reaction conditions for different processes.

CO_2_was converted to CH_4_ using Ni/CNT, Ni/SBA-15, and (Cu, Ca, Mg, Mn, Co)–Ni/SBA-15 catalysts, with Ni/SBA-15 exhibiting the highest efficiency [7]. Due to the excellent catalytic activity of nickel-based catalysts at relatively moderate temperatures, they were chosen for organic solvent reformulation, hydrogen reduction, and carbon nanotube synthesis. On the other hand, nickel was chosen for methane cracking, steam reorganization, and partial oxidation processes in the chemical industry due to its high selectivity in organic reactions and demonstrated ability and availability to break C-H and C-C bonds [8,9,10,11].

Iron-grafted SBA-15 has been proven as a sustainable, efficient, heterogeneous, and regio-selectivity catalyst for direct arylation of biphenyl methane and benzene [12], reducing aromatic nitro compounds to form aromatic amines [13] and the benzylation reaction of benzene with benzoyl chloride [14]. Furthermore, Fe/SBA-15 catalysts could be effective in performing the Fenton reaction to decompose sodium formate and an organic copper-containing dye in water [15]. Fe species in the mesoporous framework material are tetrahedrally coordinated and turn to iron oxide after high-temperature calcination [16]. Iron-containing SBA-15 was determined for the characteristics of ironic catalytic materials [17], conducted to remove phenolic aqueous solutions [18], and was used to develop magnetic material for drug delivery in the medicine industry [19].In addition, metal oxide-incorporated mesoporous SBA-15 materials were employed as a non-enzymatic sensing device for glucose detection, and the Cu-SBA-15-modified electrode presented electro-catalytic activity toward the oxidation of glucose [20].

According to Ni-based catalyst systems, results indicated highly efficient conversion to increase the stability of catalysts for methane reforming. Nickel catalysts stabilize inside the mesoporous SBA to reduce the opportunity for sintering and coke deposition, protecting the metal nanoparticles [21,22,23,24]. Co catalysts could also reduce coke deposition in many hydrocarbon conversions and present the advantage of high-activity Co-based catalysts [25,26,27,28,29,30]. Fe, Co, and Ni are commonly selected as catalysts to enhance the different carbon sources for the formation of carbon nanomaterials [8,31,32]. In addition, nickel- and cobalt oxides-impregnated SBA-15 materials presented resistance to coke formation, with high activity and selectivity, and were applied to organic solvents (cyclohexane, n-hexane, propane, and CO) [33] and biomass-derived fuels (such as liquid hydrocarbons and diesel-range hydrocarbons) [34,35], the growth of carbon nanofibers [36], bio-oil to produce hydrogen and carbon nanotubes [37], and n-hexane, propane and CO oxidation [38]. Some literature indicated higher performance in converting methyl esters to produce green diesel (C11-C20 hydrocarbons) [39]. Ni/SBA–15 was more stable than Ni/Al_2_O_3_ to reform guaiacol at 600 °C [40], and it also presented high efficiency in the application of the dry reforming of methane [41] to enhance CO_2_ reforming of CH_4_ [42].

Therefore, this study examined the detailed properties of SBA-15 and metal oxides impregnated with SBA-15, specifically iron, cobalt, and nickel oxides. In this context, the physicochemical properties of SBA-15 and Fe, Co, and Ni oxides impregnated withSBA-15 were characterized using scanning electron microscopy (SEM), transmission electron microscopy (TEM), energy dispersive spectroscopy (EDS), pore characteristic analysis, and X-ray diffraction (XRD).In addition, isopropanol was selected to decompose on SBA-15 and metal oxide-impregnated SBA-15 to evaluate the application of mesoporous materials and the performance of metal catalysts.

## 2. Materials and Methods

### 2.1. Material Preparation

1. Santa Barbara Amorphous (SBA-15) synthesis

SBA-15, a mesoporous material, was synthesized with triblock copolymers to present a two-dimensional hexagonal symmetry structure. The method for synthesizing SBA-15 was adapted from past studies [1,2,43]. The 4.0 g of amphiphilic tri-block copolymer surfactant ((poly(ethylene glycol)-block-poly(propylene glycol)-block-poly(ethylene glycol), PEG-PPG-PEG), EO20PO70EO20) (Pluronic P123, molecular weight: 5800, Aldrich, St. Louis, MO, USA) was weighed and thoroughly dissolved into the 2M HCl solution, followed by 1hour of mixing. Next, 6.4 g of tetraethyl orthosilicate from a silica source (TEOS, Aldrich, St. Louis, MO, USA) was weighed and added into the reaction mixture, which was then mixed at 30 °C for 24 h. Following that, the reactant mixture was mixed for 24 h at a temperature of 90 °C.

The filtration technique was used to collect the mixture’s solid products. Next, the particles were washed with distilled water and air-dried. The dried solid products were calcined at 500 °C in the air for 5 h in order to remove the structure-directing agent from them. This was followed by smashing the solids into the SBA-15 powder once they had cooled to room temperature.

2. Impregnation ofmetal oxides on SBA-15

Ferric nitrate nonahydrate(Fe(NO_3_)_3_·9H_2_O, J.T. Baker, Phillipsburg, NJ, USA),cobalt nitrate hexahydrate(Co(NO_3_)_2_·6H_2_O, Alfa Aesar, Heysham, England), and nickel nitrate hexahydrate (Ni(NO_3_)_2_·6H_2_O, J.T. Baker, Phillipsburg, NJ, USA) were dissolved in the distilled water solution and mixed with SBA-15 powder to prepare metal-impregnated SBA-15 at concentrations of 2, 5, and 8 wt%. The metal nitrate solutions and SBA-15 mixture were mixed for 30 min with a so nicator before the metal oxides-impregnated SBA-15 particles were filtered out at room temperature. In order to remove any remaining water, the particles were dried at 60 °C in an oven. After drying, the metal oxides-impregnated SBA-15 particles were calcined at 550 °C for 5 h at a rate of 5 °C/min in an electric thermal furnace under an argon atmosphere. This procedure was designed to produce 2–8 wt% metal-SBA-15.

### 2.2. Characterization of Materials

1. Scanning electron microscopy (SEM)

The morphology of SBA-15 and metal oxides-SBA-15 materials was examined using a field emission SEM (JEOL JSM-6700F, Peabody, MA, USA) functioning at 1–2 kV.

2. Transmission electron microscopy (TEM)

A high-resolution transmission electron microscope (HR-TEM, JEOL JEM-2010, Tokyo, Japan) with a 200kV accelerating voltage was used to examine the material structure.

3. Pore structure characteristics

All of the material’s physical properties (specific surface area, microporous area, total pore volume, microporous volume, and pore diameter) were measured with N_2_ (gas) adsorption/desorption at 77 K in a pore characteristic analyzer (ASAP 2010, Micromeritrics Inc., Norcross, GA, USA).The surface area was calculated using the BET method; the micropore surface area was determined by subtracting the external surface area from the BET surface area [44]; total pore volume was determined by the desorption branch of the BJH method [45]; and micropore volume was calculated by the t-plot and the Harkins–Jura method [46,47]. Silica-alumina, alumina, and molecular sieve from Micromeritrics were selected as standard materials for quality assurance and quality control.

### 2.3. Chemical Compositions

1. Energy Dispersive X-ray Spectrometry (EDS)

The surface composition of the material samples, including elements C, N, O, Si, Ni, and Cl, was investigated using an SEM (JXA-840, JEOL, Japan) equipped with an energy dispersive X-ray spectrometer (AN10000/85S, Links, England). For the purposes of quality assurance and control, a total of five samples were analyzed in duplicate.

2. X-ray Diffraction(XRD) Analysis

To identify the crystalline process, two powder X-ray diffractometers with the characteristics of Cu-K radiation (=0.15406 nm) and their scintillation detector were used. The scanning ranges were 0–10 (small-angle X-ray scattering (SAXS) analysis, Bruker AXS Gmbh, Karlsruhe, Germany) and 10–80 (XRD, TTRAX-III, Rigaku, Japan), with 0.02 step, and 1 sec/step.

### 2.4. Carbon Material Formation on SBA-15 and Metal-Impregnated SBA-15

1. Carbon source preparation

In order to reduce the template of SBA-15, Fe-SBA-15, Co-SBA-15, and Ni-SBA-15, hydrogen gas was injected into the chemical vapor deposition furnace at a flow rate of 200 mL/min in an environment of 450 °C for 1 h. Isopropanol (IPA), as the carbon source, was added into the purge tubes at a constant temperature of 2.0 ± 0.1 °C in a temperature-controlled water chamber. The saturated IPA vapor with a concentration of 1.03 ± 0.12% was produced, while ultra-pure nitrogen was applied as the purge gas, and the concentration was determined using gas chromatography with a flame ionization detector. Furthermore, the amount of IPA which was influent into the reaction furnace was determined and checked by using a balance.

IPA/N_2_ gas flowed into the CVD furnace at a rate of 50 mL/min for 30 min at various temperatures (600, 700, and 800 °C). IPA broke down on the SBA-15, Fe-SBA-15, Co-SBA-15, and Ni-SBA-15 templates to form carbon materials. The carbon materials formed on the SBA-15 template were referred to as SBA-15-600C, SBA-15-700C, and SBA-15-800C for IPA breakdown temperatures of 600, 700, and 800 °C, respectively. Regarding the IPA breakdown, the metal oxide-impregnated templates M-SBA-15 (M is referred to as Fe, Co, and Ni oxides) were referred to as M-SBA-15-600C, M-SBA-15-700C, and M-SBA-15-800C at the various temperatures.

2. Mass fraction of carbon materials

The amount of carbon material formed on the template and liquid products (tar and soot) was determined with a weight balance (Mettler, XSR205DU).

The experimental process involved measuring the weight difference in a stove, placing the weighted templates into crucibles (*W_i_*), and drying the samples in an oven at a fixed temperature of around 150 °C for 4 h;the weight difference should be under 5 mg. Then, the weighted template crucible was placed into the furnace for the experiment. After the experiment, the crucible was weighed after carbon material formation on templates (*W_f_*). The influent IPA concentration was determined by using GC-FID. The IPA influent concentration and flow rate were employed to determine the weight of IPA (*W_IPA_*) in the furnace.

$$W\left(\%\right)=\frac{W_{f}-W_{i}}{W_{IPA}}\times 100$$

where *W* is the fraction of carbon material; *W_i_* is the initial weight of the template; *W_f_* represents the weight of the sample after carbon material formation on the template after the experiment, and *W_IPA_* is the IPA weight.

## 3. Results and Discussion

### 3.1. Morphology of Materials

**Figure 1:**
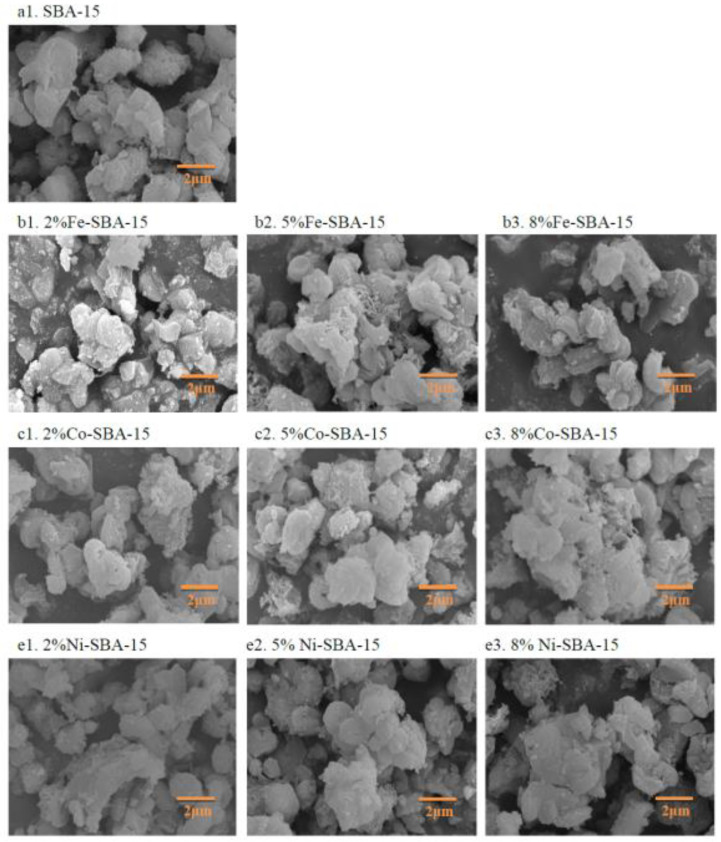
SEM morphology of SBA-15 and Fe-, Co-, and Ni-impregnated (2–8 wt%) SBA-15.

For SBA-15, the average particle sizes were 3.1 ± 1.4 μm (in the range of 1.2–4.9 μm) according to the SEM photos (Figure 1). The iron-impregnated SBA-15 particle sizes were 2.9 ± 0.8 μm for 2% Fe-SBA-15, 2.4 ± 0.6 μm for 5% Fe-SBA-15, and 3.2 ± 0.8 μm for 8% Fe-SBA-15. The average particle sizes were 3.7 ± 1.3 μm for 2% Co-SBA-15, 3.0 ± 1.2 μm for 5% Co-SBA-15, and 3.6 ± 1.4 μm for 8% Co-SBA-15. In addition, the average particle sizes were 2.9 ± 1.7, 4.5 ± 1.2, and 4.2 ± 1.3 μm for 2% Ni-SBA-15, 5% Ni-SBA-15, and 8% Ni-SBA-15, respectively. There were insignificant differences in average particle sizes between SBA-15 and metal (2–8 wt% of Fe, Co, and Ni)oxides-doped SBA-15 particles by SEM photos.

The literature indicated thatSBA-15 materials are rod-like particles, and their length aspect could be several to hundred μm, with a material diameter within μm [48,49]. The silica source and the hydrolysis products associated with the silica source could both influence the morphology of SBA-15. The rod-like SBA-15 was formed under the coexisting conditions of tetramethyl orthosilicate in the amphiphilic triblock copolymer P123 [49], which was similar to the results of this research.

**Figure 2:**
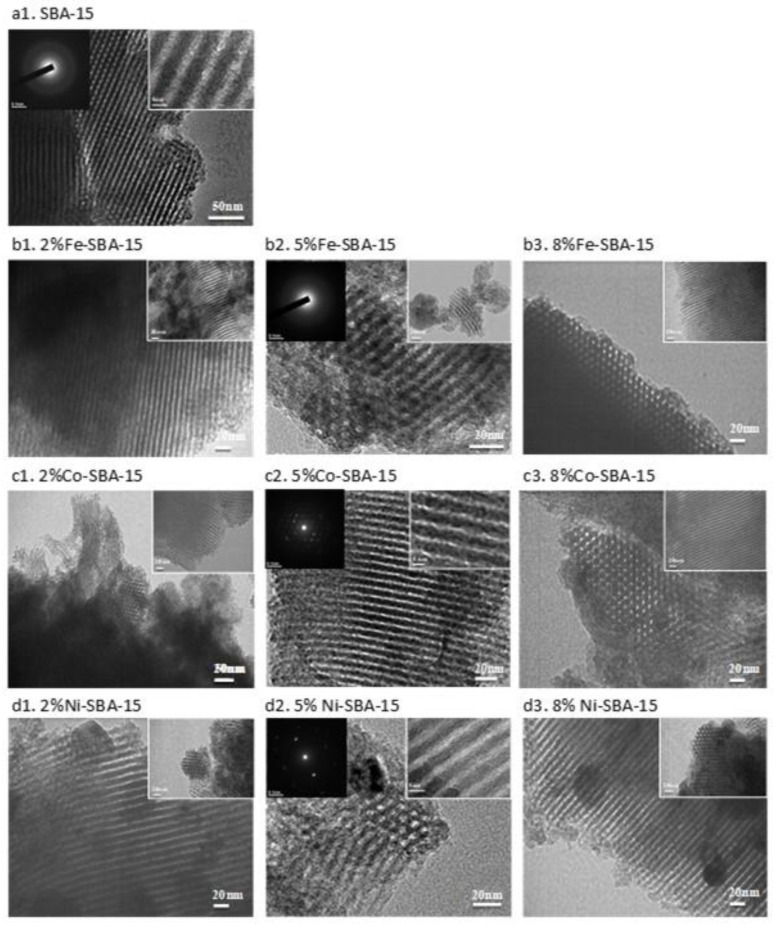
TEM morphology of SBA-15 and Fe-, Co-, and Ni-impregnated (2–8 wt%) SBA-15.

For TEM analysis, the uniform pore structure characteristics and regular shape were determined in SBA-15 (with a pore diameter of about 5.2–6.8 nm (the average wall thickness was 2.5 ± 0.5 nm (range from 1.7 to 3.2nm), shown in Figure 2), and a sturdy wall maintained structural stability while in use. Using TEM image analysis, we were able to determine that the SBA-15 pores had diameters in the range of 7.5 ± 0.3 nm and a thickness of 2.5 ± 0.3 nm, respectively [50]. Both our previous research and the current TEM imaging performed in this study confirmed that the templates were thermostable and capable of maintaining a hexagonal pore shape under temperatures ranging from 650 to 950 degrees Celsius for up to 2 h. [51].As the calcination temperature rose, decreases in the total pore volume, micropore volume, and mean pore size of SBA-15 were observed [52], which were similar to this study. It also reflected the stable thermal characteristics of SBA-15. For the TEM-SADP(selected area diffraction patterns) photo, there was no crystal determination in SBA-15.

For iron-impregnated SBA-15 (Figure 2b), the pore diameters were 5.5, 4.9, and 4.5 nm for 2, 5, and 8 wt% Fe-SBA-15, respectively. The high iron doping concentration seemed to reduce the pore diameter of SBA-15. After metal impregnation, the pore diameter and structure of SBA-15 were found to be similar to those of neat SBA-15. The wall thicknesses of Fe-SBA-15 were 3.2, 3.6, and 3.6 nm for 2, 5, and 8% Fe-impregnated SBA-15, respectively.

For cobalt-impregnated SBA-15(Figure 2c), results indicated the pore diameters could be 5.0, 5.5, and 4.2 nm for 2, 5, and 8 wt% Co-SBA-15, respectively. The wall thicknesses of Co-SBA-15 were 4.2, 3.0, and 3.0 nm for 2, 5, and 8% Co-impregnated SBA-15, respectively.

For nickel-impregnated SBA-15(Figure 2d), results indicated the pore diameters could be 6.5, 4.3, and 6.0 nm for 2, 5, and 8 wt% Ni-SBA-15, respectively. The wall thicknesses of Ni-SBA-15 were 2.3, 3.1, and 2.7 nm for 2, 5, and 8% Ni-impregnated SBA-15, respectively. Nickel particles were depicted as small black dots on the image of Ni-SBA-15 (Figure 2), ranging in size from 28 to 56 nm for different nickel impregnation concentrations.

Metal oxides impregnated on SBA-15 seemed to increase the pore wall thickness, especially in the Co and Fe doping. The pore diameter of metal oxide-impregnated SBA-15 may be reduced in comparison with the parent SBA-15. Metal oxide impregnation and high-temperature calcination could be the reasons. However, the hexagonal cylinder and mesoporous structures of SBA-15 did not significantly change after the metal oxide impregnation procedures.

### 3.2. Nitrogen Isotherms and Pore Size Distribution

**Figure 3:**
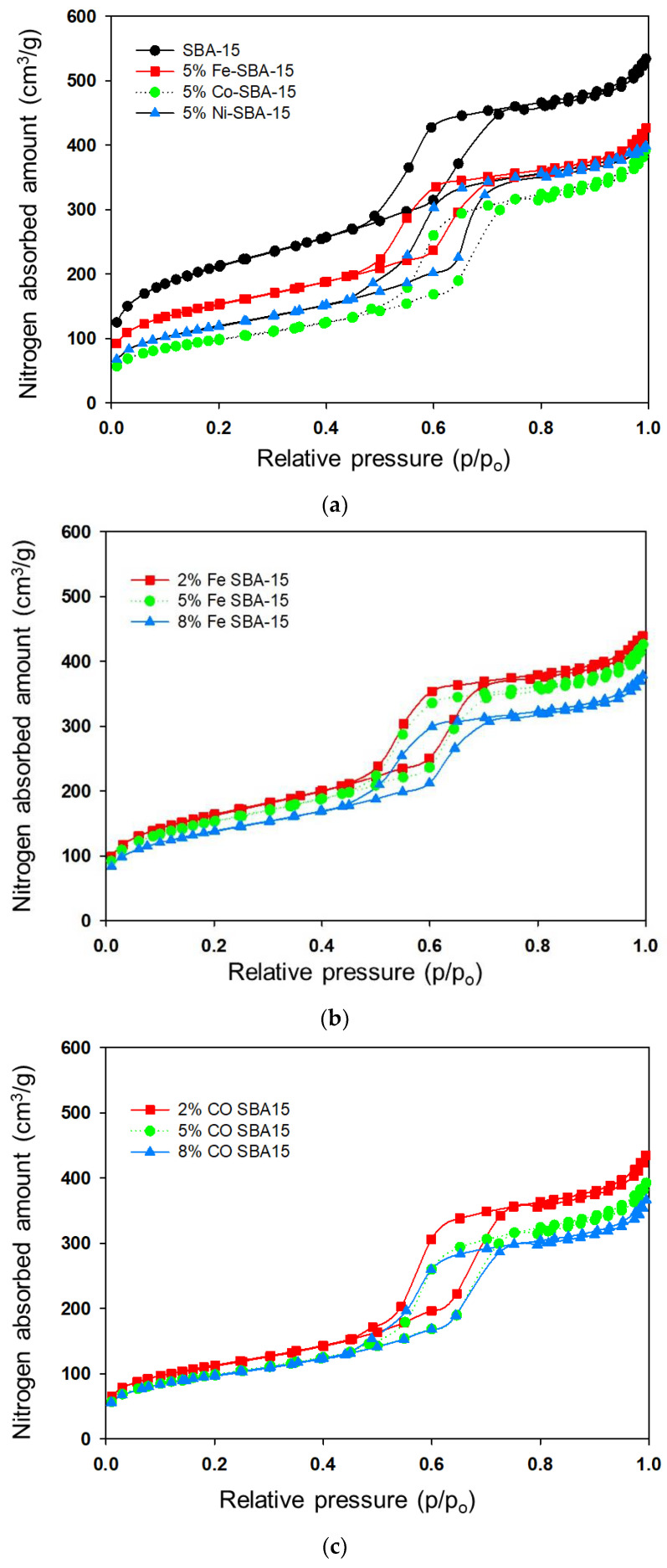

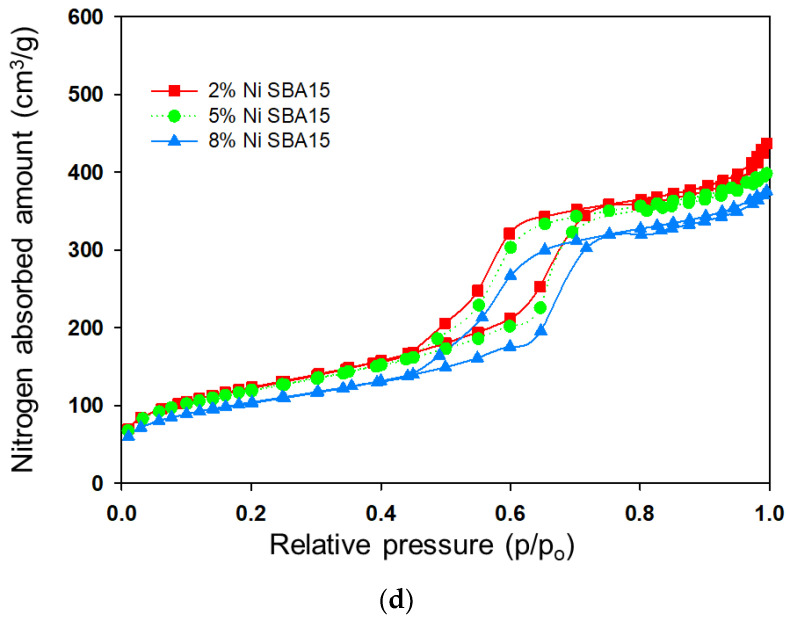
(**a**) Nitrogen adsorption isotherms of SBA-15 and 5 wt% (Fe, Co, Ni)-SBA-15. (**b**) Nitrogen adsorption isotherms of 2–8 wt% of Fe-SBA-15. (**c**) Nitrogen adsorption isotherms of 2–8 wt% of Co-SBA-15. (**d**) Nitrogen adsorption isotherms of 2–8 wt% of Ni-SBA-15.

The nitrogen adsorption–desorption isotherms of SBA-15, Fe-SBA-15, Co-SBA-15, and Ni-SBA-15 are shown in Figure 3. The typical type IV adsorption isotherms for the SBA-15 and metal-impregnated SBA-15 were determined, and all of these materials exhibited H1-type hysteresis and revealed cylindrical channels. Results point out the blocking effect on the materials during the nitrogen sorption procedures, and the large metal doping aggregate pore structures were attributed to the effects.

Five duplicated samples were determined for their pore characteristics. The specific surface area was 667 ± 51 m^2^/g for the SBA-15, and the specific surface area could be reduced by 14–26%, 44–49%, and 36–45% after iron-, cobalt-, and nickel-impregnated SBA-15 to form Fe-SBA-15, Co-SBA-15, and Ni-SBA-15, respectively (shown in Table 1). The literature indicated the SBA-15 template presented systematic stability after the impregnation and calcination procedures [4,25,53,54].

According to the nitrogen adsorption isotherm, the SBA-15 revealed monolayer adsorption on the walls of the mesopores at the lower relative pressure (P/P_0_ < 0.40). At relative pressures in the vicinity of 0.50, capillary condensation was observed in the isotherms as the relative pressure increased. On the other hand, multilayer adsorption of SBA-15 was observed at higher relative pressures. Sharp nitrogen adsorption at a relative pressure in the vicinity of 0.6–0.7 corresponded to the well-uniform mesoporous structures of SBA-15 that were similar to those of other studies [55,56]. After the metal impregnation ofSBA-15, the sharp nitrogen adsorption zone could be moved to a lower relative pressure because the metal impregnation changed the textural structures of support.

**Figure 4:**
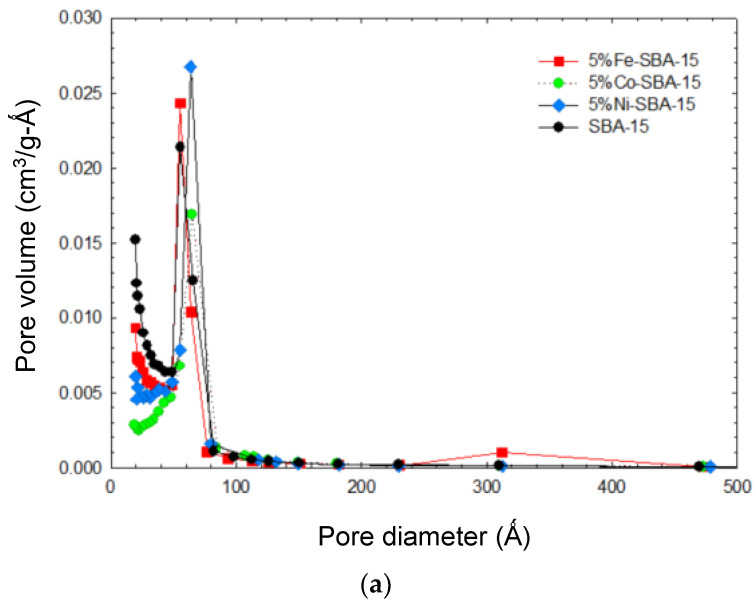

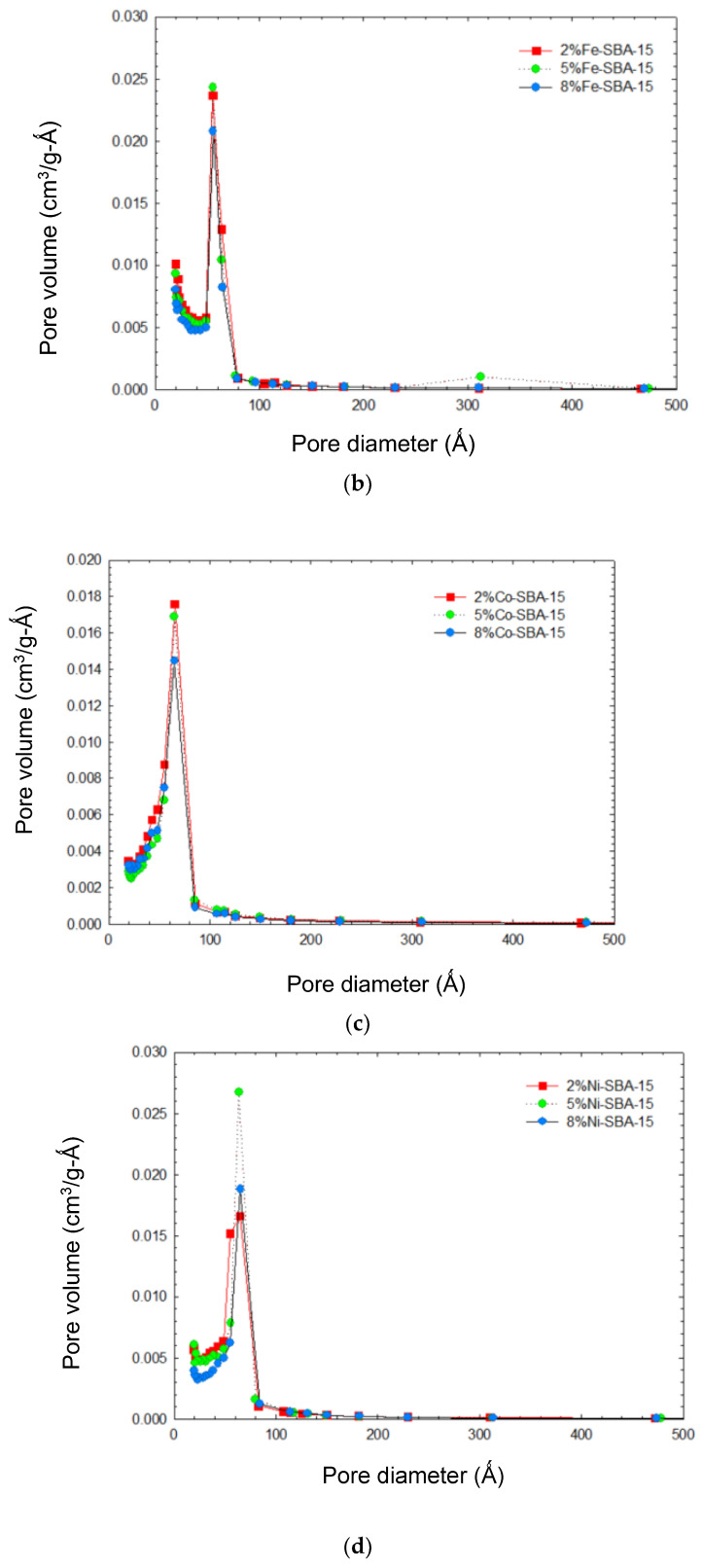
(**a**) Pore size distribution of SBA-15and 5 wt% (Fe, Co, Ni)-SBA-15. (**b**) Pore size distribution of 2–8 wt% of Fe-SBA-15. (**c**) Pore size distribution of 2–8 wt% of Co-SBA-15. (**d**) Pore size distribution of 2–8 wt% of Ni-SBA-15.

The average pore diameter of SBA-15 was 45.7 Å, and that of Fe-SBA-15 was 47.5–48.5 Å, Co-SAB-15 was 65.2–66.8 Å, and Ni-SAB-15 was 59.9–64.4 Å. This could have been caused by a partial blockage of the SBA-15 pores by cobalt oxide clusters and/or a partial collapse of the mesoporous structure, both of which resulted in an increase in pore diameter after metal impregnation [57,58]. Metal additives on SBA-15 have the potential to block pores and increase the average diameter of the pores. There was a 20–30% decrease in pore volume after metal oxides were impregnated on SBA-15. Metals additives were found to block the pores in the micro-and meso-ranges of SBA-15 and cause an increase in the average pore diameter. The pore volume reduction in metal-impregnated SBA-15 was at a pore diameter of less than 50 Ǻ (shown in Figure 4) compared with SBA-15. However, the Fe-impregnated SBA-15 presented a relatively high surface area and pore volume compared with both Co- and Ni-impregnated SBA-15. The decrease in the pore volume of Co-SBA-15 and Ni-SBA-15 in comparison with Fe-SBA-15 could have been caused by the blocking of the pores by the metal clusters. In addition, the low pH value (pH was from 2.4 to 1.9 corresponding to the 2 to 8% of Fe(NO_3_)_3_ solution) of the Fe(NO_3_)_3_ solution enhanced the reaction with SBA-15 particles that may have led to pore development, especially in mesopores (shown as Figure 4). According to the pore distribution, the pore volume was high in the pore diameter range of 50–60 Ǻ and the vicinity of 300Ǻ.

For the metals impregnated into SBA-15, the micropore surface was reduced for both Co- and Ni-impregnated SBA-15 for different metal loading, but the Fe-doped SBA-15 was insignificant in its reduction in micropore surface area. Although the metal oxides deposited on the surface of SBA-15 reduced the surface area, more new pores could be developedinFe-SBA-15 than in both Co- and Ni- SBA-15.

Ni that was impregnated on other silica mesoporous materials had a similar effect in increasing the pore diameter for silica mesoporous materials [59]. There was a decrease in the surface area of the impregnated 2–8 wt% metals(Fe, Co, and Ni) on SBA-15; this could be associated with either pore blockage or the loss of crystallites. The metal sites on SBA-15 were uniformly distributed for loadings ranging from 2 to 8 weight percent metals. In addition, SBA-15 surface impregnation with cobalt and nickel particles was identified using transmission electron microscopy (TEM). The results showed that the structures of SBA-15, Fe-SBA-15, Co-SBA-15, and Ni-SBA-15 were all similar to one another. Some black dot points were separated on the Co-SBA-15 and Ni-SBA-15, which could have been the cobalt and nickel, and the metal oxide crystals were also reflected by the results of TEM-SADP photos.

### 3.3. Energy Diffraction Spectra (EDS)

Table 2 shows the major elemental constituents of SBA-15 and metal-impregnated SBA-15, as determined by EDS. SBA-15 was primarily composed of silica (60%) and oxygen (40%). The silica contents reduced from 56 to 42%, and oxygen increased from 42 to 50%, corresponding to the 2% to 8% iron-impregnated SBA-15. For cobalt-impregnated SBA-15, the silica decreased from 53 to 42%, and oxygen increased from 45 to 50%, corresponding to the 2% to 8% cobalt-impregnated SBA-15. For Ni-SBA-15, the silica decreased from 44 to 38%, corresponding to the 2% to 8% nickel-impregnated SBA-15. However, the oxygen did not depend on the metal impregnation concentration. The oxygen abundance was in the range 52–54%. The average metal oxide content in impregnated SBA-15 was 2.1/5.6/9.4, 2.4/6.9/10.9, and 1.9/6.8/10.5% at 2/5/8 %wt for iron-, cobalt-, and nickel oxides-impregnated SBA-15, respectively.

### 3.4. X-ray Diffraction (XRD)

**Figure 5:**
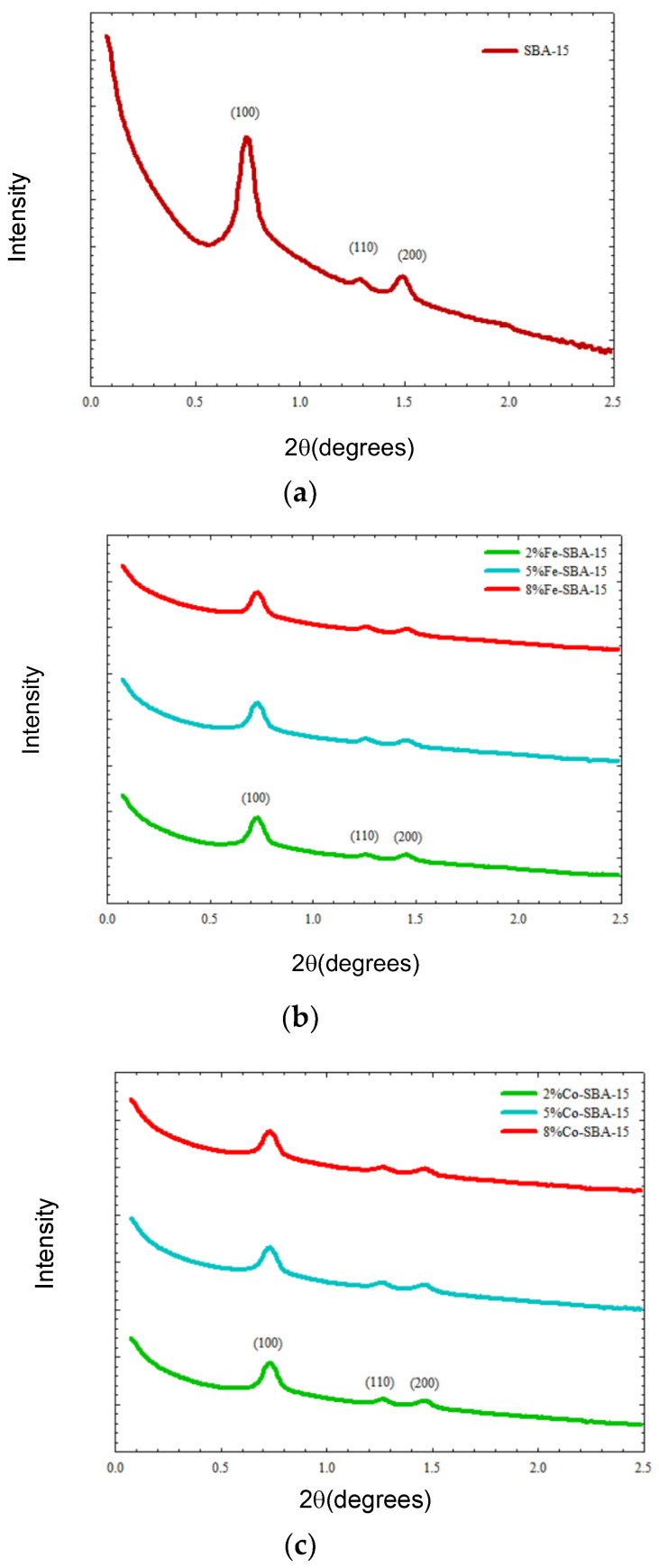

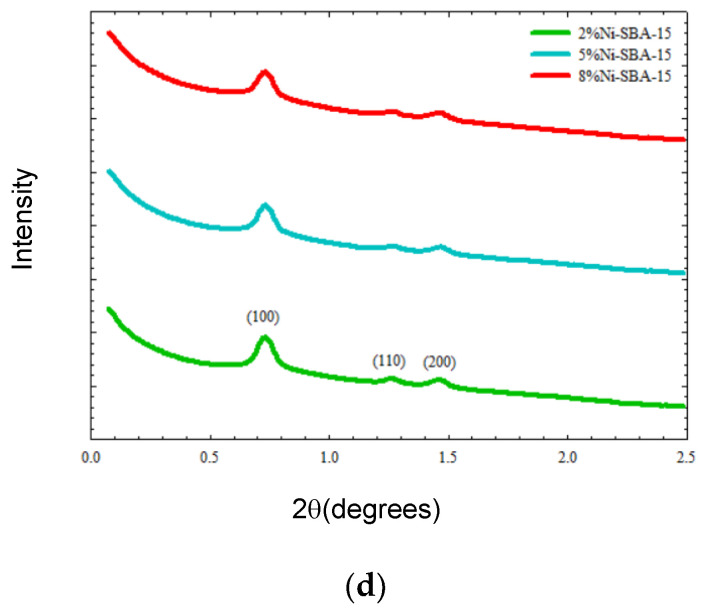
(**a**) SAXS analysis of SBA-15. (**b**) SAXS analysis of 2–8 wt% of Fe-SBA-15. (**c**) SAXS analysis of 2–8 wt% of Co-SBA-15. (**d**) SAXS analysis of 2–8 wt% of Ni-SBA-15.

Figure 1 shows bundled rope-like structures for SBA-15 and metal-impregnated SBA-15 (Fe-SBA-15, Co-SBA-15, and Ni-SBA-15). Based on small-angle X-ray diffraction (Figure 5), there were three peaks determined for the SBA-15 lattice as (100), (110), and (200) at 2θ in the vicinity of 0.7, 1.2, and 1.4, respectively. There were three well-resolved diffraction peaks, which corresponded to the hexagonally ordered structure of SBA-15 [60].The metal oxide-doped materials also indicated the ordered hexagonal mesoporous structure but lowered the intensity of peaks after impregnation. Small-angle XRD was used to determine the two-dimensional hexagonal structure diffraction of SBA-15 and its metal oxide-doped materials (intensive peak at 2θ = 0.6–0.8) (Figure 5).

**Figure 6:**
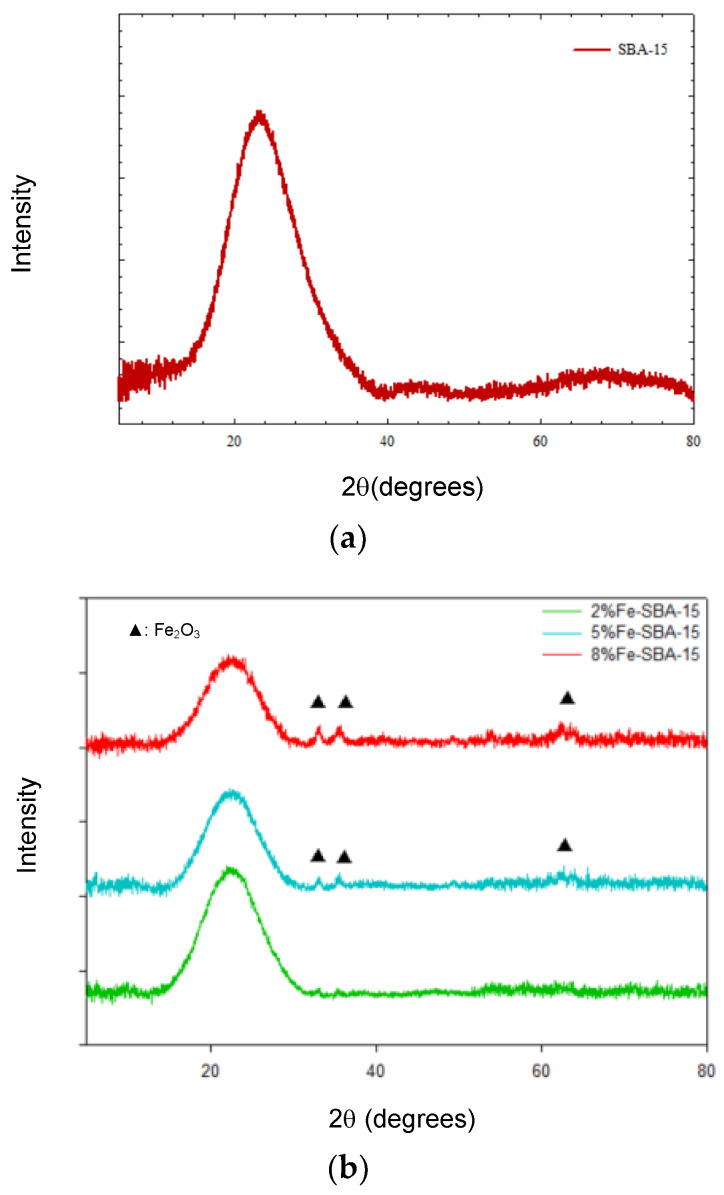

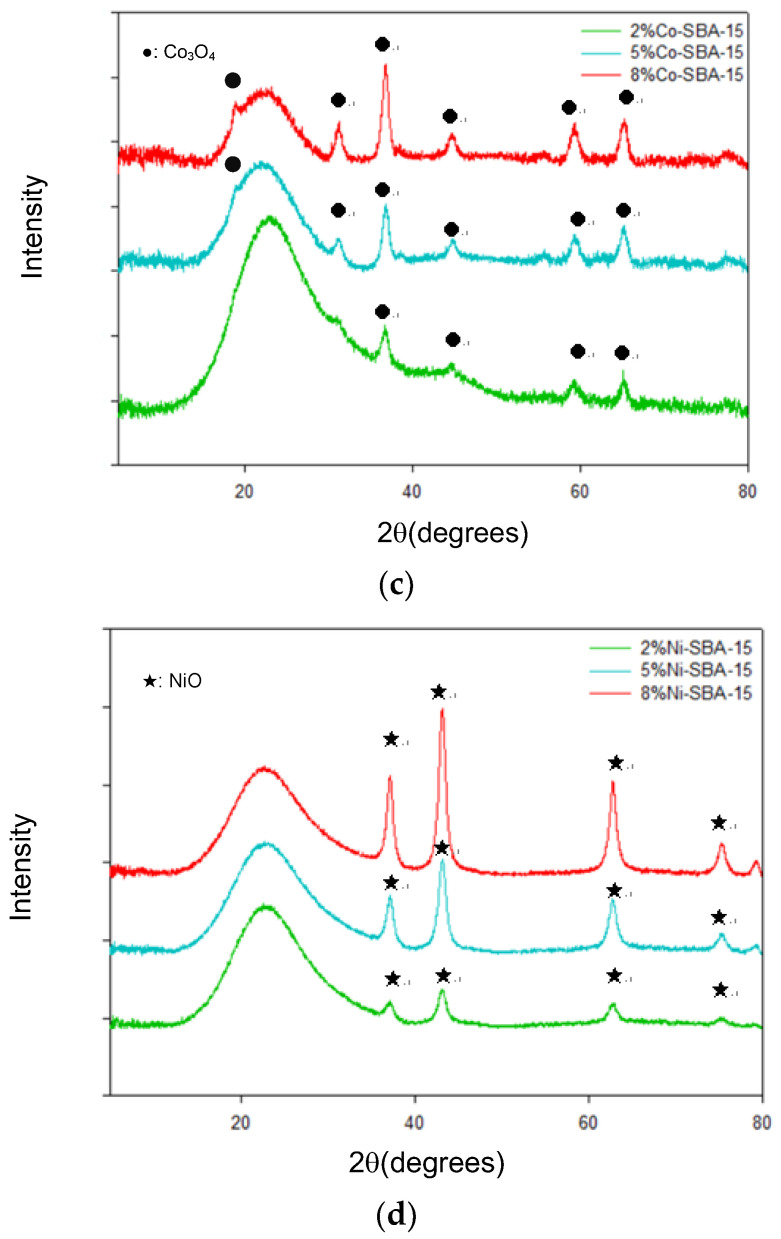
(**a**) XRD analysis of SBA-15. (**b**) XRD analysis of 2–8 wt% of Fe-SBA-15 (▲: Fe_2_O_3_). (**c**) XRD analysis of 2–8 wt% of Co-SBA-15 (●:Co_3_O_4_). (**d**) XRD analysis of 2–8 wt% of Ni-SBA-15 (★:NiO).

SBA-15 and metal oxide-impregnated SBA-15 exhibited a broad silica peak near 23.8°, indicating the amorphous silica on the support (Figure 6).

Three main Fe_2_O_3_ diffraction peaks were determined at 2θ=33.6, 35.8, and 62.7° for Fe-SBA-15, corresponding to the Miller indices (104), (110), and (214), respectively; the positions of the peaks were the same as those of a previous study [61]. Lower iron loading peaks in the crystal spectrum were insignificantly displayed by XRD. While the iron loading was over 5%, the small iron crystal peak spectrum determined by XRD was similar to that of other studies. The crystal peaks were presented under high Fe loadings via XRD, but the crystal peaks were insignificant for low Fe loading (3–6%) [62]. The Fe_2_O_3_grain sizes were in the range of 13.7–30.3 nm, according to Scherrer’s equation.

Five main Co_3_O_4_ diffraction peaks were determined at 2θ=31.3, 36.9, 44.8, 59.4, and 65.2°for Co-SBA-15, corresponding to the Miller indices (220), (311), (400),(511),and (440), respectively, and the peak positions of XRD were similar to those in other literature [63,64,65]. In addition, one weak Co_3_O_4_ diffraction peak was observed in the vicinity of 2θ=19.0 for 5 and 8% Co-SBA-15, corresponding to the Miller index (111). The grain sizes of Co_3_O_4_ were in the range of 11.2–79.1 nm, and the high metal loadings presented high grain sizes.

Four main NiO diffraction peaks were determined at 2θ = 37.2, 43.2, 62.8, and 75.4° for Ni-SBA-15, corresponding to the Miller indices (111), (200), (220), and (311), respectively (shown in Figure 6d); the results were similar to those of other studies [66,67]. The grain sizes of NiO on SBA-15 were in the range of 9.2–11.8 nm through the determination of the XRD spectrum and Scherrer’s equation.

Generally, high metal oxide loading revealed high grain sizes of metal oxide crystals. According to the XRD spectrum and Scherrer’s equation, the sequence of average grain size of metals on SBA-15 was Co-SBA-15(11.2–79.1 nm)> Fe-SBA-15 (13.7–30.3 nm)> Ni-SBA-15 (9.2–11.8 nm).

The testing of the application of these materials was a limitation of this study. The detailed physicochemical characteristics of metal-doped SBA-15 and raw materials presented baseline information for their applications in the future. These materials can be used as catalyst templates for organic vapor decompositions to form carbon materials such as carbon nanotubes and for the reformation of organic waste solvents to produce high-value materials. In addition, metal oxide-impregnated SBA-15 could be used for the decomposition of organic solvents, bio-oil reforming materials, and medical applications for drug delivery materials.

### 3.5. IPA Decomposed on SBA-15 and Metal Oxide-Impregnated SBA-15

1. Mass fraction of carbon materials and residues

**Figure 7:**
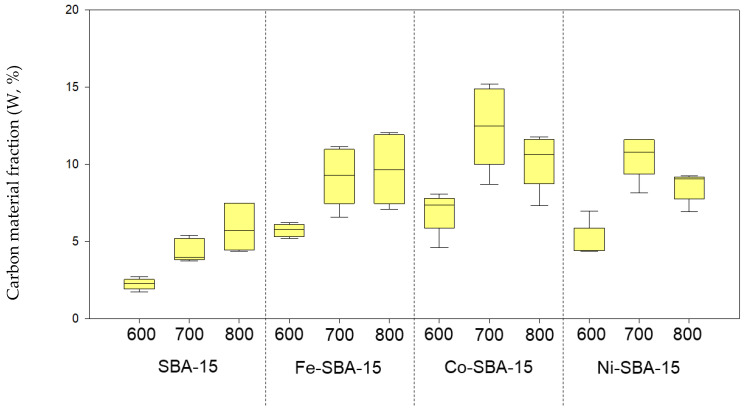
Carbon materials formation on SBA-15, and Fe-, Co-, and Ni oxides-impregnated SBA-15 at 600, 700, and 800 °C.

Two percent metal-loaded SBA-15 templates were selected to evaluate the performance of IPA decomposition. Based on the mass fractions of IPA decomposed at various temperatures, little IPA was found to decompose on the template and form carbon materials. However, there was more carbon material formation on metal oxide-impregnated Ni-SBA-15 and Fe-SBA-15 than on SBA-15. Almost 90% of IPA was formed as tar, soot, and exhaust in the liquid and gas phases (Figure 7). Due to the exhaust gas constituents, some weight loss occurred. However, this problem was not addressed directly in this study.

As the temperature increased from 600 to 800 °C, the formation of carbon materials increased from 2.2 to 5.9%, while tar and residues decreased from 46 to 22% for IPA decomposed on SBA-15. Moreover, no carbon filaments were observed on SBA-15. When IPA was decomposed on Fe-SBA-15, Co-SBA-15, and Ni-SBA-15 under this temperature, carbon material formation increased from 5.7 to 9.7, 6.9 to 12.4, and 5.0 to 10.5% with the increase in temperature, respectively. The tar and residues were in the range of 41–61, 44–50, and 46–56%, respectively. The results pointed out that the fraction of tar and residues was not dependent on the reaction temperature. The carbon materials formed on metal-impregnated SBA-15 could be 2–3 times that of the amount of carbon materials formed on SBA-15 at 600–800 °C. Although high temperatures can improve the decomposition of IPA to form carbon materials on the support and reduce the fraction of tar and residues, it may lead to the vaporization of the liquid products.

2. Carbon materials

**Figure 8:**
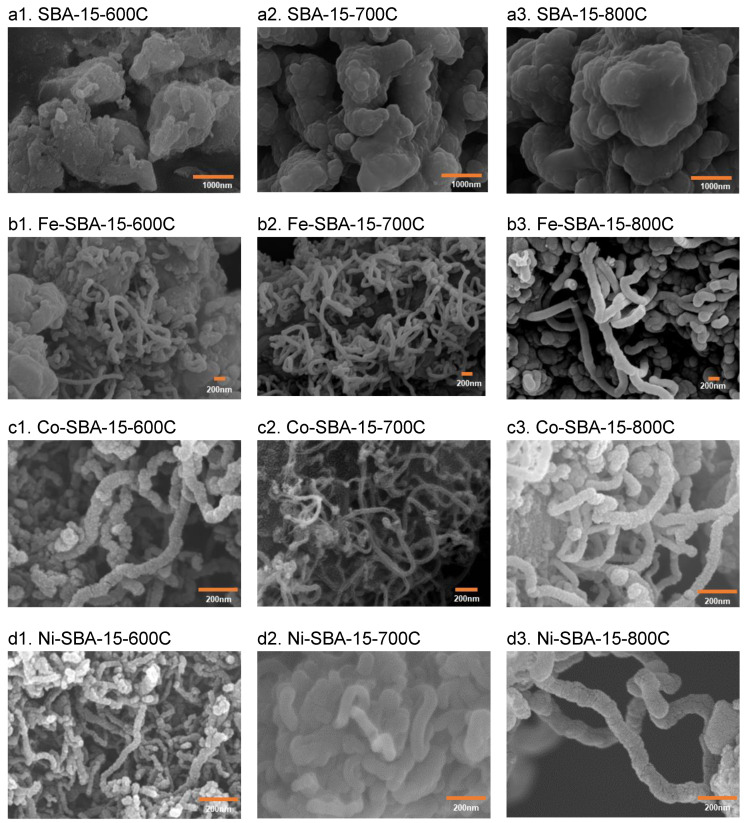
Carbon material formation on SBA-15 and Fe-, Co-, and Ni-SBA-15 after IPA decomposition at 600, 700, and 800 °C.

After the experiments were conducted under temperatures of 600–800 °C on SBA-15, none of the formations of carbon filaments could be found through SEM analysis (Figure 8). The results showed that the IPA was decomposed to form soot or coke on SBA-15. 

The diameter of the filaments was in the range of 70–100 nm on Fe-SBA-15 at 600 °C. At 700 °C, carbon tubes with diameters ranging from 65–100 nm formed. At 800 °C, the diameters of the carbon tubes were larger (up to 140–180 nm) than those of the materials constructed at 600–700 °C. The diameters of the filaments were 53–70, 40–65, and 46–70 nm on Co-SBA-15 at 600, 700, and 800 °C, respectively. The diameters of the filaments were 35–60 nm on Ni-SBA-15 at 600 °C. At 700 °C, carbon tubes with diameters ranging from 50–100 nm formed. At 800 °C, the diameters of the carbon tubes were larger (up to 80–110 nm) than those of the materials formed at 700 °C.

## 4. Conclusions

SBA-15 is a two-dimensional hexagonal and mesoporous structure silica material. Its specific surface area could be reduced by 14–26%, 44–49%, and 36–45% after 2–8% of iron, cobalt, and nickel oxides were impregnated on SBA-15, respectively. The small-angle powder XRD patterns of SBA-15 materials exhibited three peaks in the 2θ range, 0.71–1.45°, which corresponded to the diffractions of 100, 110, and 200 planes, respectively. These peaks presented the characteristic hexagonally ordered structure of SBA-15 materials. Metal oxides impregnated on SBA-15 appeared to increase the pore wall thickness, while decreasing the pore diameter when compared to the SBA-15. The metal-doped materials also showed that the ordered hexagonal mesoporous structures of support and metal loading did not change the hexagonal structure of the initial matrix. According to the XRD spectrum analysis, Fe_2_O_3_, Co_3_O_4_, and NiO were the crystalline structure on the Fe-SBA-15, Co-SBA-15, and Ni-SBA-15, respectively. Metal oxides-impregnated SBA-15 can be employed as the catalyst for pollution removal in different media, organic transformation materials, and waste recycling into high-value materials.

## Figures and Tables

**Table 1:** Pore characteristics of SBA-15 and Fe-, Co-, and Ni-impregnated (2–8 wt%) SBA-15.

Materials	BET Surface Area (m^2^/g)	PV (cm^3^/g)	Micropore Surface Area (m^2^/g)	MPV (cm^3^/g)	PD (Å)
SBA-15	666.7	0.83	97.4	0.052	45.7
2% Fe-SBA-15	571.3	0.68	91.7	0.048	47.5
5% Fe-SBA-15	552.8	0.67	90.4	0.047	48.5
8% Fe-SBA-15	494.1	0.59	85.7	0.044	47.8
2% Co-SBA-15	387.4	0.63	38.7	0.019	65.2
5% Co-SBA-15	375.2	0.62	39.2	0.019	66.8
8% Co-SBA-15	350.1	0.57	32.6	0.016	65.6
2% Ni-SBA-15	427.7	0.64	26.1	0.012	59.9
5% Ni-SBA-15	411.8	0.63	33.3	0.015	60.9
8% Ni-SBA-15	362.7	0.58	35.7	0.017	64.4

Note: PV: average pore volume; MPV: micropore volume; PD: average pore diameter.

**Table 2:** EDS analysis of SBA-15 and Fe-, Co-, and Ni oxides-impregnated (2–8 wt%) SBA-15.

Materials	Si (wt%)	O (wt%)	Metal Oxide (wt%)
SBA-15	60.3 ± 1.28	39.7 ± 1.28	-
2% Fe-SBA-15	56.07 ± 8.43	42.10 ± 8.51	2.14 ± 0.14
5% Fe-SBA-15	49.47 ± 7.48	45.67 ± 7.67	5.57 ± 0.42
8% Fe-SBA-15	42.1 ± 1.16	49.7 ± 1.1	9.41 ± 0.53
2% Co-SBA-15	53.27 ± 5.92	44.87 ± 6.18	2.45 ± 0.57
5% Co-SBA-15	49.97 ± 15.5	44.93 ± 15.56	6.91 ± 0.79
8% Co-SBA-15	42.37 ± 5.1	49.63 ± 5.26	10.9 ± 0.80
2% Ni-SBA-15	44.1 ± 2.49	54.43 ± 2.5	1.87 ± 0.06
5%Ni-SBA-15	41.93 ± 0.97	52.03 ± 1.07	6.83 ± 0.37
8% Ni-SBA-15	37.5 ± 1.76	54.27 ± 1.27	10.5 ± 0.84

## Data Availability

Not applicable.
